# Species richness declines and biotic homogenisation have slowed down for NW-European pollinators and plants

**DOI:** 10.1111/ele.12121

**Published:** 2013-05-21

**Authors:** Luísa Gigante Carvalheiro, William E Kunin, Petr Keil, Jesus Aguirre-Gutiérrez, Willem Nicolaas Ellis, Richard Fox, Quentin Groom, Stephan Hennekens, Wouter Landuyt, Dirk Maes, Frank Meutter, Denis Michez, Pierre Rasmont, Baudewijn Ode, Simon Geoffrey Potts, Menno Reemer, Stuart Paul Masson Roberts, Joop Schaminée, Michiel F WallisDeVries, Jacobus Christiaan Biesmeijer

**Affiliations:** 1 School of Biology, University of LeedsLS2 9JT, Leeds, UK; 2 Naturalis Biodiversity CenterP.O. Box 9517, 2300RA, Leiden, The Netherlands; 3 Department of Ecology and Evolutionary Biology, Yale University165 Prospect Street, New Haven, CT, 06520, USA; 4 Center for Theoretical Study, Charles University and the Academy of Sciences of the Czech RepublicJilska 1, 110 00, Praha 1, Czech Republic; 5 Working Group Lepidoptera Faunistics, Zoological Museum, Section EntomologyPlantage Middenlaan 64, 1018 DH, Amsterdam, The Netherlands; 6 Butterfly ConservationManor Yard, East Lulworth, Dorset, BH20 5QP, UK; 7 Botanical Society of the British Isles, c/o Botany Department, British Museum (Nat. Hist.)London, UK; 8 Centre for Ecosystem Studies, Wageningen University and ResearchAlterra, PO Box 47, NL-6700 AA, Wageningen, The Netherlands; 9 Research Institute for Nature and Forest (INBO)Kliniekstraat 25, 1070, Brussels, Belgium; 10 Laboratory of Aquatic Ecology and Evolutionary Biology, University of LeuvenCharles Deberiotstraat 32, B-3000, Leuven, Belgium; 11 Laboratoire de Zoologie, Université de MonsPlace du Parc 23, B-7000 Mons, Belgium; 12 FLORONPostbus 1413, 6501 BK, Nijmegen, The Netherlands; 13 School of Agriculture, Policy and Development, University of ReadingReading, RG6 6AR, UK; 14 European Invertebrate Survey–Netherlands/Naturalis Biodiversity CenterPostbus 9517, 2300 RA, Leiden, The Netherlands; 15 Institute for Water and Wetland Research, Radboud University NijmegenP.O. Box 9010, 6500 GL, Nijmegen, The Netherlands; 16 De Vlinderstichting/Dutch Butterfly ConservationP.O. Box 506, 6700AM, Wageningen, The Netherlands; 17 Laboratory of Entomology, Wageningen UniversityP.O. Box 8031, 6700 EH, Wageningen, The Netherlands; 18 University of Amsterdam, Institute of Biodiversity and Ecosystem DynamicsP.O. Box 94248, 1090 GE, Amsterdam, The Netherlands

**Keywords:** Accumulation curves, biodiversity loss, community ecology, plant–flower visitor communities, pollination, similarity, spatial homogenisation, species richness estimations, temporal and spatial patterns

## Abstract

Concern about biodiversity loss has led to increased public investment in conservation. Whereas there is a widespread perception that such initiatives have been unsuccessful, there are few quantitative tests of this perception. Here, we evaluate whether rates of biodiversity change have altered in recent decades in three European countries (Great Britain, Netherlands and Belgium) for plants and flower visiting insects. We compared four 20-year periods, comparing periods of rapid land-use intensification and natural habitat loss (1930–1990) with a period of increased conservation investment (post-1990). We found that extensive species richness loss and biotic homogenisation occurred before 1990, whereas these negative trends became substantially less accentuated during recent decades, being partially reversed for certain taxa (e.g. bees in Great Britain and Netherlands). These results highlight the potential to maintain or even restore current species assemblages (which despite past extinctions are still of great conservation value), at least in regions where large-scale land-use intensification and natural habitat loss has ceased.

Ongoing species richness loss and biotic homogenisation are a growing concern for society (Pereira *et al*. 2010; Cardinale *et al*. 2012). The increasing awareness of the role of human activities in these declines and their potential impacts on our health, food supply, ecosystem services and well being (e.g. Isbell *et al*. 2011) have led to policy changes towards the protection of biodiversity, many implemented after 1990 (Kleijn & Sutherland 2003). The Convention on Biological Diversity's main target is to substantially reduce the rate of biodiversity loss at global, regional and national levels (CBD, Secretariat of the Convention on Biological Diversity 2010), and the EU committed itself to an even more ambitious target of halting biodiversity loss. Most agree that these targets have not been met (e.g. Butchart *et al*. 2010); but in the absence of standardised long-term monitoring programmes (which exist for only a few taxa and countries) it is difficult to assess if biodiversity loss trends are changing.

Biesmeijer *et al*. (2006) use rarefaction methods to estimate changes in diversity in plant and pollinator taxa based on historical collections before and after 1980. They found evidence of declines in bees and pollinator-dependent plants in Great Britain and the Netherlands. However, whereas it is well known that during the past decades the studied countries underwent substantial climatic (EEA, European Environment Agency 2004), land-use (Haines-Young *et al*. 2003; EEA, European Environment Agency 2010; FAO, Food & Agriculture Organization of the United Nations 2012) and environmental policy changes (Kleijn & Sutherland 2003), it remains unclear how the rate of biodiversity loss has changed over time.

Focusing only on native species assemblages, here we apply robust richness estimation methods to evaluate richness changes during the past 80 years in three European countries (Great Britain, the Netherlands and Belgium; *ca*. 29.2 million records in total) at multiple spatial scales (from 10 × 10 km cells to the whole of each country), and also evaluate changes in patterns of biotic homogenisation (due to the spread of native species or loss of spatially restricted species). We find that extensive species richness loss and biotic homogenisation occurred before 1990. However, such negative trends have slowed down (or recovery has set in) for several taxa during the most recent decades.

## Materials and methods

For each of the studied countries (the United Kingdom, the Netherlands and Belgium), four 20-year time periods were compared: 1950–1969 (hereafter P1), 1970–1989 (P2), 1990–2009 (P3) and whenever possible 1930–1949 (P0), covering a period during which the studied countries were subjected to substantial changes in land-use, climate and environmental policies. The analysis of data collected prior to 1950 (1930–1949) was limited to taxonomic groups and countries for which data quality allowed this.

Three groups of flower visiting insects which contribute to plant pollination were studied here: bees (Hymenoptera: Apoidea), hoverflies (Diptera: Syrphidae) and butterflies (Lepidoptera: Papilionoidea and Hesperioidea). Given the importance of bees for pollination, and the recognised high vulnerability of bumble bees (Apidae, Bombini) (Carvell *et al*. 2007; Williams & Osborne 2009), we analyse bumble bees separately from other bees (the honeybee, *Apis mellifera* L. was not considered, as its presence in the focal region is largely dependent on apiculture practices). Plants were divided into three groups according to their dependence on flower visitors for pollination: fully dependent, intermediate dependency and independent, as described below. Exotic species (archaeophytes and neophytes) represent a relatively small minority of species, and analyses including exotics generated similar result patterns for most spatial scales; therefore, we focus only on the results obtained using data on native plant species. For details on the databases sources see Table S1.

Information on the dependency of plants on insects for pollination was obtained for *ca*. 50% of the plant species (2481 of 4943 species) from the Ecological Flora database (see Table S1). When information was not consistent among the three databases a given species was classified as intermediately dependent. On the basis of this information we detected that only 6% of the genera (23 of 377 genera, single species genera excluded) and only 35% of the families (36 of 103 multi-species families) included both insect-dependent and non–insect-dependent species. Therefore, for the *ca*. 50% of species for which information was not available in any of the databases, we used information from genus and family level to classify the species (e.g. all Poaceae were classified as pollinator independent). If species within a genus had different dependencies, species would be classified as intermediately dependent. As many tree species are planted for ornamental or reforestation purposes, we repeated the analyses with and without all tree species to distinguish between effects of natural vs. man-induced range changes. As no major differences were found between analyses with and without trees, we presented the results including all native plant species.

### Analyses of species richness change

In the absence of formal monitoring programmes, long-term databases containing validated species records collected at different times and by many different recorders provide a valuable source of information on past and present species occurrences. As these records were usually not collected following standardised sampling schemes, their analysis faces several methodological challenges. First, improvement of taxonomic and collection tools/skills through time can lead to more species being registered in more recent time periods simply due to detectability changes. To ensure that taxonomic changes and collection tools/skills would not affect estimates of richness change, species that could not be easily distinguished in the past or present time periods were lumped into aggregate species, based on information provided by the specialists on each of the taxa (Bees: DMichez, PR, SR; Hoverflies: MR, FVM, Butterflies: RF, DMaes, MW, Plants: QG, SH, WVL, BO, JS). Plant species with multiple subspecies or varieties were always aggregated under a unique name. Secondly, estimates of richness change are generally dependent on the spatial scale at which they are evaluated (Keil *et al*. 2010). However, such scale-related differences in estimates provide valuable information to help understand the patterns of change, and for conservation management (Whittaker *et al*. 2001). For example, range expansions affect richness values of multiple fine-scale cells, and hence have a substantial effect on the mean change value at finer spatial scales (Gotelli & Colwell 2001), but no effect at country level (if the species was present in both periods, and simply changed its distribution pattern). Conversely, country-level extinctions of spatially restricted species and species introductions will affect coarse (e.g. national)-scale richness, while only influencing a few fine-scale cells (see Cassey *et al*. 2006). Therefore, whenever data quality allowed, we repeated richness change analyses at multiple spatial scales (e.g. 10 × 10, 20 × 20, 40 × 40, 80 × 80 or 160 × 160 km grid cells as well as for the whole country). As only one 160 × 160 km grid was available for the Netherlands and Belgium, this scale was not considered in these countries. Finally, unequal sampling intensity between grid cells or time periods and oversampling of rare species can bias richness estimates (Hellmann & Fowler 1999; Garcillan & Ezcurra 2011; ter Steege *et al*. 2011). Here, we combine robust richness estimation methods with a meta-analysis approach to deal with the unstandardised nature of historical collections.

When comparing two periods with unequal sampling, previous studies have used rarefaction to estimate (or rather, interpolate) the value of richness in the more sampled period that would be expected if sampling effort had been equal to that of the less sampled period (e.g. Biesmeijer *et al*. 2006; Keil *et al*. 2010). As an alternative, Colwell *et al*. (2012) have shown that species accumulation curves can be extrapolated (up to threefold of the real sampling effort), producing reasonable estimates of richness further in the accumulation curve. This approach has been used here to estimate richness of the least-sampled period, extrapolating to a point where sampling effort is equal between the two periods (pre-period *X*_1_[*n*] and post-period *X*_2_[*n*]). However, where the more sampled period had more than threefold the number of records of the less sampled period, we combined extrapolation and interpolation in our comparisons: extrapolating the smaller sample's accumulation curve up to three times its sample size, and rarifying the larger sample down to this same ‘comparison point’.

We repeat this process for every cell, calculating relative richness change between the pre- and the post-period as 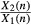. For analyses of richness change, we applied a log transformation (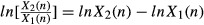, hereafter termed ‘logratio’) to normalise residuals. Its sampling variance (VAR_*QX*_) is approximated as 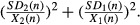 As in the absence of singletons, the assemblage is considered to be well sampled, and SD of *X* will be zero (Colwell *et al*. 2012), to account for any under- or overestimation of singletons and doubletons (collectors may put more effort on registering the rarest species, Garcillan & Ezcurra 2011, or try to obtain two specimens to capture morphological diversity of the species, e.g. males and females; flowering and fruiting plant specimens), we *a priori* excluded cells with very poor quality of sampling and we used bootstrapping to calculate SD of *X* (by re-sampling the data we create a corrected estimator of the number of unseen species, where the effect of under/oversampling of singletons is reduced). For details on selection criteria applied in richness change analyses, see legend of Table S2.

Meta-analysis techniques were then applied to obtain an overall weighted value of richness change (*Qw*) and assess if the mean value of change at a given scale was significantly different from zero. Using the rma.uni function of the R package metaphor (Viechtbauer 2010), each grid cell was weighted based on the inverse of the variance, so that cells with more reliable estimations have a higher weight in the analyses (Hartung *et al*. 2008). To check if the method completely corrected for bias due to differences in sampling efforts, we included the log of the relative difference in the number of records between the two time periods 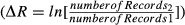 as a covariate. If accumulation curve estimations did not completely remove the bias due to sampling effort (i.e. whenever ΔR had a significant effect on estimated richness change across sites), we calculated the partial residuals after removing the effect of sampling effort for each cell to obtain unbiased estimates of richness change for each grid cell. We then assessed in which cells richness had significantly increased or decreased using a *z*-test, for each species group, country and time period. Finally, we evaluated if values of richness change were spatially autocorrelated by comparing a model with spatial autocorrelation structure (linear or exponential) with a null model using a log-likelihood ratio test and compared the Akaike Information Criterion values of each model.

For verification of the sensitivity of our results, we repeated all richness analyses using only interpolation and only extrapolation and we compare the results using re-sampled and non–re-sampled SD and variance when analysing data.

### Analyses of species assemblage similarity through time and space

To evaluate changes in patterns of biotic homogenisation, we investigate how similarity (and thus turnover) of species assemblages across space (evaluated by comparing assemblages in 10-km grid cells) changes over time. Classical measures of assemblage similarity or dissimilarity (beta-diversity, e.g. Sorensen similarity or Jaccard similarity) can be partitioned into two components: dissimilarity due to species replacement and dissimilarity due to nestedness (see Baselga 2010). Here, we use similarity due to species replacement (calculated as 1-Beta-sim, Lennon *et al*. 2001), a measure that is independent of, but complementary to, our measurements of alpha-diversity (see Baselga 2010).

To correct for the unequal sampling effort, for each group and each 20-year time period, we used an individual-based probabilistic approach (Chao *et al*. 2005) for calculating similarity in species composition between each selected grid cell (focal cell) and all other selected grid cells, not allowing for repetition of pairs of grid cells. As species identity is crucial for similarity analyses, we applied selection criteria stricter than for the richness analyses (see details on selection criteria in the legend of Table S3). Applying less strict selection criteria would lead to the selection of more grid cells, but could lead to results more influenced by cells which due to their low sample size have only the commonest species.

We then estimated the distance decay of similarity (logit transformed to standardise residuals) at the different time periods. We used a general linear-mixed model (with focal grid cell as a random factor and distance and time period as fixed variables) fitted using maximum likelihood (estimates obtained using restricted maximum likelihood) using the R package nlme (Pinheiro *et al*. 2012). As in richness analyses, the probability of a given species being selected in a random sample of records will depend on the evenness of records per species. As evenness of records per species decreases with increasing sampling effort (Table S4), the probability of an assemblage being a nested subset of another (such that 1-Beta-sim = 1) is likely to increase with increasing difference in sampling effort between the two grid cells. This effect is, however, likely to be less accentuated when the least-sampled cell is already very well sampled. To test and correct for this effect, the number of records of the least-sampled grid cell, the relative difference of number of records between the two grid cells and the interaction between the two were included in the model as explanatory terms.

All calculations described above were made using scripts written by LGC, PK and Tom van Dooren for R (R Development Core Team 2012), and scripts can be provided upon request to LGC.

## Results

**Figure 1:**
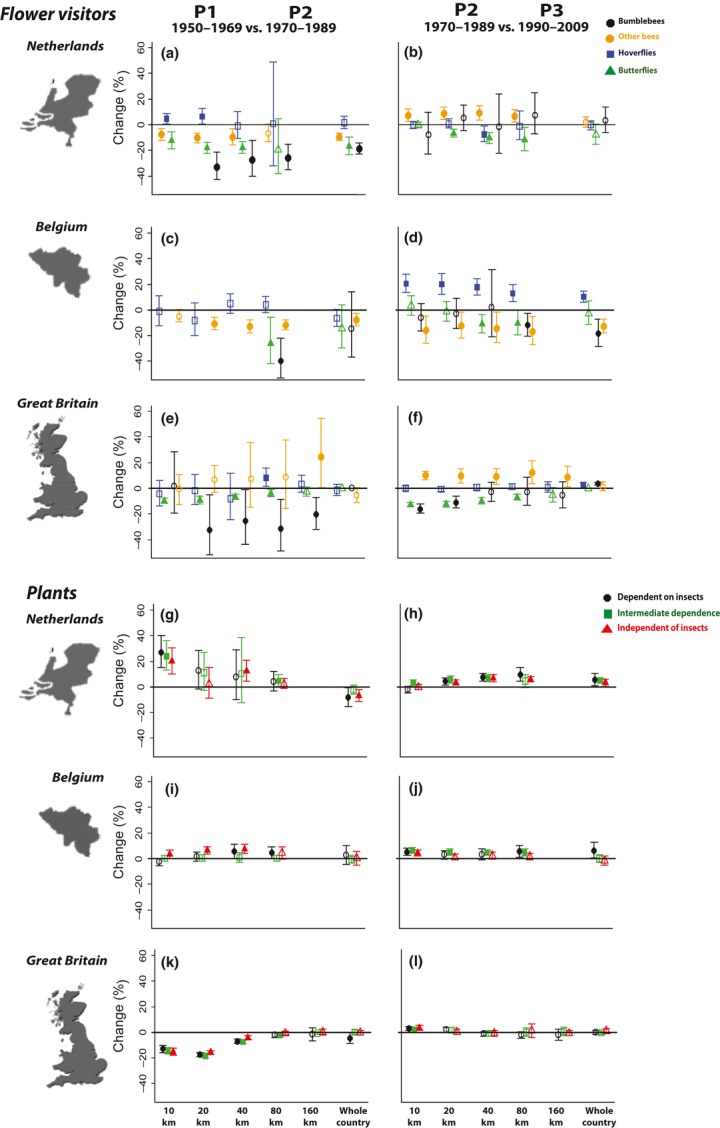
Change in species richness (estimated weighted mean ± 95% confidence intervals) of flower visitors and plants through time at different spatial scales. For most taxa and countries richness change estimates (% of change) of flower visitors and plants were more accentuated between P1 and P2 (the Netherlands, a, g, Belgium, c, i, and Great Britain, e, k) than between P2 and P3 (the Netherlands, b, h, Belgium, d, j, and Great Britain, f, l). Due to insufficient number of grid cells, results from some spatial scales are not presented for some groups. The horizontal line represents no change (0%). Filled symbols indicate that change was significantly different from zero, otherwise symbols are open (see statistical details in Table S2).

Whereas analyses at coarser spatial scales (e.g. country level) were always possible, the number of finer spatial scale locations that matched our selection criteria varied between taxa and countries (Fig. S1). Although evenness of records per species significantly changed with sampling effort and time period (Table S4, which affect richness change estimations per cell), overall we found consistent patterns of average native richness change per spatial scale when using interpolation combined with extrapolation (Fig. 1, Fig. S2), only interpolation (Fig. S3) or only extrapolation (Fig. S4), and when using different weights in the meta-analyses (Table S5). Overall richness change values for each 10-km cell are provided for each taxonomic group and country (Fig. S5) to display of variability in richness change values and to assess how our methodological approach corrected for differences in sampling effort between time periods. When comparing 1970–1989 vs. 1990–2009, significant spatial autocorrelation was detected for all taxa in Great Britain (plants, butterflies, hoverflies and bees), for Dutch plants and hoverflies and for Belgium hoverflies. For Great Britain, significant spatial autocorrelation was also detected when comparing 1950–1969 vs. 1970–1989 (Table S6). The degree of autocorrelation did not undermine subsequent calculation of mean values of change at different spatial scales.

### Changes in flower visitor and plant species assemblages up to 1990

**Figure 2:**
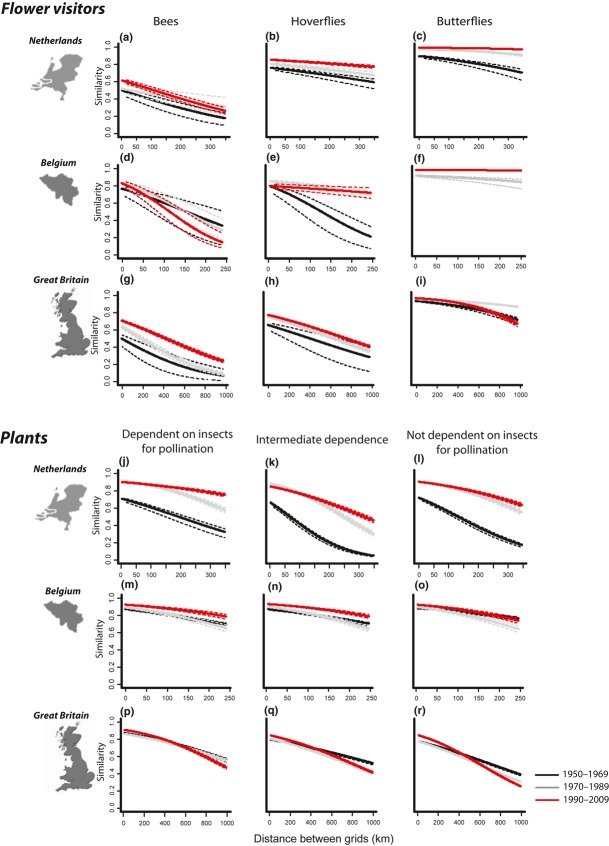
Changes in species compositional similarity (1-βsim) of plants and flower visitors among 10-km grid cells through space and time. Decline in similarity with distance is presented for bees, hoverflies, butterflies, plants dependent on insects for pollination, plants of intermediate dependence and plants independent of insects for pollination of the Netherlands (a, b, c, j, k, l, respectively), Belgium (d, e, f, m, n, o) and Great Britain (g, h, i, p, q, r). Strong patterns of spatial homogenisation (i.e. increases in similarity) were detected between P1 and P2 (a, c, e, i, j, k, l). Lines represent the estimated value of similarity ± 95% confidence intervals, after removing the effect of sampling effort (for statistical details see Table S3). Data were insufficient for butterflies in Belgium for time period P1.

Patterns of species assemblage change differed between taxa, countries and time periods. When comparing 1950–1969 (P1) and 1970–1989 (P2), substantial richness changes were noted in all insect groups. Butterfly richness fell significantly in all three countries, and at almost all spatial scales (Fig. 1a,c,e). Substantial declines in bees were also detected between P1 and P2 in the Netherlands and Belgium (Fig. 1a,c). In Great Britain, at the few locations for which available data were sufficient for the comparison of these two time periods (see Fig. S1.5), declines were also found in bumble bee species richness, whereas the richness of other bees did not change significantly at most scales, even showing a tendency to increase at relatively coarse scales (Fig. 1e). However, when looking further back in time (i.e. comparing P0 and P1), richness declines were found for both bee groups in all three countries (in Great Britain significant only at the coarsest scale) (see Fig. S2). Moreover, the spatial homogeneity of bee community species composition increased significantly in Great Britain between P1 and P2 (Fig. 2g), suggesting that any increases in richness in non–bumble bees were driven by expansion of common species. Significant spatial homogenisation of species assemblages was also detected in the Netherlands for bees and butterflies between P1 and P2 (Fig. 2a,c). Hoverflies fared better over this period, with no significant richness declines being detected in any of the countries (Fig. 1a,c,e), and slight increases at fine spatial scales in the Netherlands (Fig. 1a), but with significant increases in homogeneity across space (Fig. 2,b,e,h).

For plant assemblages, between P1 and P2, only British native species richness declined significantly at finer spatial resolutions (10–40 km; Fig. 1k). No such losses were apparent for the plants of the Netherlands or Belgium over this period, but in the Netherlands richness declines were detected at the coarsest resolution (i.e. whole country, Fig. 1g), which may be associated with loss of species that due to their restricted ranges do not have a major influence on the average values of finer scales. In the Netherlands, we even detected significant increases in plant richness at finer (10–40 km) spatial resolutions (Fig. 1g). This increase at local scales, however, was accompanied by spatial homogenisation of native communities across space (Fig. 2j,k,i), which suggests that in the Netherlands locally unique species were replaced by native widespread species (for patterns of change in exotic plants, see Fig. S6). Plants displayed qualitatively similar patterns of change between these periods (P1 vs. P2) regardless of their dependence on pollinators (see Figs 1 and 2).

### Changes in flower visitor and plant species assemblages since 1990

Over more recent decades (P2: 1970–1989 vs. P3: 1990–2009) declines in species richness or spatial heterogeneity slowed down for many of the studied taxa and countries (see Figs. 1 and 2). In the Netherlands and in Great Britain, bumble bee richness declines became less accentuated, whereas species richness of other bees increased significantly at fine and intermediate scales in both Great Britain and the Netherlands (Fig. 1b,f). Bumble bee richness increases were also detected in Great Britain at country level (Fig. 1f), which is associated with the well-documented arrival of *Bombus hypnorum* L. (Goulson & Williams 2001). Furthermore, no further spatial homogenisation of communities was detected for bees in the Netherlands (Fig. 2a). Dutch butterfly richness declines also became less accentuated at finer scales (with richness increases detected at the finest spatial scales in the Netherlands). Only for Belgian bees and British butterflies was the magnitude of richness decline maintained over this period, even becoming more accentuated at some spatial scales (Fig. 1d,f). Although butterfly assemblage homogeneity was high in all three countries in P2, significant increases were still detected in Belgium and the Netherlands (Fig. 2c,f), whereas in Great Britain a marked increase in biotic heterogeneity was detected (possibly caused by range contractions of some previously widespread species) (Fig. 2i). For hoverflies, richness increases were detected at all spatial scales in Belgium, whereas in Great Britain and the Netherlands no significant changes were found over this period. The process of spatial homogenisation essentially stopped for this group (Fig. 2), with only weak increases in the Netherlands and Great Britain.

Patterns of plant diversity change (richness and similarity) were also much less marked between the more recent periods P2 and P3 (Figs. and 1 and 2), with rates of diversity change slowing in both the Netherlands (Fig. 1h, see also Fig. S7) and in Great Britain (Fig. 1i), where richness declines were no longer detected. Indeed, a partial recovery of species richness was detected in the Netherlands at coarse scales, and in Great Britain at finer scales. Homogenisation trends in the Netherlands also became less accentuated (Fig. 2j,k,l). In Belgium, mild richness increases at finer scales continued to be detected (Fig. 1j) and increases in spatial homogeneity became more accentuated (Fig. 2m,n,o).

## Discussion

Evaluating how rates of biodiversity loss are changing through time and space is essential to better understand the role of the different potential drivers and the effectiveness of mitigation measures. Although rates of change have been assessed for a few taxa for which standardised monitoring schemes have been running for several decades (Van Dyck *et al*. 2009; Brereton *et al*. 2011), our combination of data sources and novel methods allows, for the first time, a detailed assessment of shifts in the strength and nature of biodiversity change for several sets of functionally linked taxa. Our findings are encouraging, indicating that declines have slowed or even been partially reversed in many groups and sites in recent decades. Although current species communities may be less diverse than in the past, they still contain many species of considerable value for conservation (see Table S1). As such, although we may not be able to reverse species extinctions, the trends here presented help justify the continuation of investment in conservation.

### Comparison with previous studies

Our analyses cover a range of plant and insect taxa across three countries, but depend on rather indirect methods for inferring dynamics. However, for taxa, regions and time periods where more direct surveys have been performed, they largely corroborate our findings, suggesting that our analytic approaches are robust. Both butterflies (Thomas *et al*. 2004; Brereton *et al*. 2011; de Vries *et al*. #b^1001^) and bumble bees (Rasmont *et al*. 2005; Williams & Osborne 2009) have been the subject of recent research, and the previously documented declines in these groups are reflected here (Fig. 1). More specifically, the more accentuated rates of decline detected for Great Britain butterflies in the most recent decades were also detected by a long-term monitoring scheme (Brereton *et al*. 2011). Previous studies also confirm our finding that a large number of non–bumble bee species have declined in Belgium after the 1950s (Jauker *et al*. 2009). For plants, data from long-term standardised surveys in Great Britain have shown that earlier declines in local plant richness in many habitats have become far less accentuated after the 1990s (Countryside Survey 2007), corroborating our results. Although no such standardised analyses were available for the Netherlands or Belgium, previous studies have reported both expansions of nitrophilous plants and loss of other species in the Netherlands during P1 and P2 (Tamis *et al*. 2005). The strong spatial homogenisation and accentuated fine-scale increases in richness found in our study for the Netherlands (Figs. 1g and 2j,k,l) are hence likely to be due to a few expanding species. Similar increases in nitrophilous plants have been noted in Great Britain (Maskell *et al*. 2010; see also Smart *et al*. 2005) and in Belgium (EEA, European Environmental Agency 2012). In Great Britain, however, the declines at finer scales detected by our study suggest that any local increases in richness between P1 and P2 were outweighed by losses of other plants, but not between earlier periods when richness increased at finer scales (see Great Britain results for 1930–1950 in Fig. S2).

Earlier comparison of pre- and post-1980 data on NW-European plant-pollinator dynamics (Biesmeijer *et al*. 2006) showed declines in the species richness of both bees and pollinator-dependent plants, suggesting a potential causal connection between plant and pollinator losses. However, our results suggest that changes are not parallel in time: although richness declines were detected in most groups at some point, they occurred in distinct time periods (Fig. 1, Fig. S1). Differences in intensity of the drivers of change through time and space (Haines-Young *et al*. 2003; EEA, European Environment Agency 2004, 2010; FAO, Food & Agriculture Organization of the United Nations 2012) and differences in ecological sensitivity of the different taxa (Reemer 2005; Carvell *et al*. 2007; Shreeve *et al*. 2001) may partly explain the diversity in patterns of change observed. Also, the different groups analysed here vary in taxonomic scope from tribe level (Bombini) to superfamilies (Lepidoptera: Papilionidae and Hersperioidea). It is possible that, similarly to what we report for the bees (Bombini vs. other bees), within each group of plants, hoverflies or butterflies, certain tribes or families will have different patterns of change than others, particularly if such tribes or families have traits (e.g. dispersal ability, diet or habitat specialisation) that may affect species susceptibility to environmental changes. More detailed data analyses involving species traits are needed to understand how species assemblage composition has changed in terms of species traits.

### What has changed for plant–flower visitor assemblages after the 1990s?

The majority of the losses in richness (bees and butterflies) and increases in homogenisation (e.g. plants in the Netherlands) that were detected between P1 and P2 (i.e. up to the 1990) slowed down between P2 and P3 (after 1990). Some insect groups even became more diverse at finer scales (e.g. non–bumble bees in the Netherlands and Great Britain, hoverflies in Belgium) in recent decades. Range expansions of some generalist species which were previously restricted (e.g. poleward shifts likely driven by climate warming after the 1980s, Parmesan *et al*. 1999) could be masking declines in more specialised species. However, in most groups, homogenisation patterns also became less accentuated concurrently, indicating that loss of locally unique species has diminished. For some groups (e.g. butterflies), homogenisation levels were already so high in P2 that further increases in P3 were constrained, but for other groups that were still spatially heterogeneous in P2 (e.g. Dutch plants, Great Britain bees), homogenisation processes between P2 and P3 were far less accentuated than between P1 and P2. In species richness terms, only butterflies (Belgium, Great Britain) and some Belgian bees showed declines in recent decades as marked as the changes between P1 and P2. Differences in environmental conditions, land-use change and in timing of the implementation of management actions that aim to protect biodiversity (e.g. agri-environmental schemes) may explain why some regions are performing better than others in terms of reducing or reversing diversity losses. For example, whereas certain drivers have likely impacted all three countries similarly (e.g. temperature increases, see EEA, European Environment Agency 2004), patterns of eutrophication, insecticide use and loss of semi-natural habitat diverged substantially between the countries (Haines-Young *et al*. 2003; Van Eetvelde & Antrop 2009; EEA, European Environment Agency 2010; FAO, Food & Agriculture Organization of the United Nations 2012). As such drivers of change are likely to be geographically explicit, and patterns of richness change likely depend on regional diversity, the significant spatial autocorrelation detected for some taxa and countries (Table S6) was expected. The lack of significant spatial autocorrelation for other taxa and time periods is likely caused by a very restricted and climatically homogeneous spatial range (e.g. plants and butterflies analyses in Belgium were restricted to Flanders) or to low number of spatial locations with good quality data (e.g. bees in Great Britain for the P1 vs. P2 comparison); although it may also be caused by drivers of change (e.g. land conversion) operating at finer scales than the ones here studied. Future detailed analysis involving spatially explicit data on changes in drivers (land use, climate, nitrogen deposition, etc.) and biodiversity may help disentangle the relative contribution of these drivers.

Our study shows that haphazardly collected data can be used for assessing changes in biodiversity loss rates. However, when sampling effort is low (e.g. Belgian butterflies or Great Britain bee comparisons at finer scales between P1 and P2), extraction of meaningful patterns is sometimes impossible. Moreover, however, sophisticated our methods, analyses of haphazardly collected data sets remain vulnerable to shifts in recorder behaviour over time. Therefore, further investment in long-term fine-scale monitoring schemes (ideally with standardised methods, modelled on, e.g. the butterfly, van Swaay *et al*. 2008) is essential to further evaluate how multiple drivers are affecting biodiversity throughout the world. Such monitoring would also allow assessment of abundance trends (Van Dyck *et al*. 2009) which cannot be detected using species accumulation methods. Such information is essential for adequate planning of conservation management and assessment of ecosystem service provision (e.g. crop pollination). Nevertheless, the deceleration of diversity losses demonstrated here suggests that declines in abundance and function may also be moderating.

The deceleration of biodiversity losses observed could be thought to be due to the species assemblages remaining in these countries being composed mostly of fairly resilient species, ones that can tolerate or benefit from anthropogenic disturbances. However, current species assemblages still bear a substantial number of rare species, habitat and food specialists, many having a recognised vulnerability and high value for conservation (see Table S1). It is, therefore, essential that future studies evaluate how variable are such trends among other taxa and regions; and that the positive trends here presented are used to motivate further research exploring the actual contribution of each driver (e.g. land-use or climate change) or specific policies (e.g. pesticide use regulation, agri-environmental schemes) to the improved change trajectories.

### Concluding remarks

Europe has some of the world's most intensively managed landscapes, but in recent decades cropland expansion has decelerated and even been partially reversed throughout large parts of the continent (EEA, European Environmental Agency 2012). Moreover, increased public awareness of the consequences of biodiversity loss has led to increased investment in measures to counteract the most negative impacts of industrial pollution (EEA, European Environment Agency 2011), habitat destruction and agricultural intensification (EEA, European Environment Agency 2010). Furthermore, farm payments have led to conversion of cropland into restored conservation or agri-environmental management areas (Kleijn & Sutherland 2003; EEA, European Environment Agency 2010). For such substantial investments to be continued, we need evidence to assess their effectiveness. Our work helps fill that gap. While we document declines in species richness and increases in biotic homogenisation in most groups and taxa in the mid 20th century, during earlier periods of accentuated loss of natural habitat (Haines-Young *et al*. 2003; Van Eetvelde & Antrop 2009; EEA, European Environment Agency 2010; FAO, Food & Agriculture Organization of the United Nations 2012), and of less investment in conservation (Kleijn & Sutherland 2003), we find strong indications that many of these problems have been ameliorated in the most recent two decades since 1990. The species assemblages remaining in these countries will likely continue to bear the marks of past declines for a long time; yet they remain diverse and contain considerable numbers of specialist and rare species. Thus, while other drivers may also contribute to the increase in richness in comparatively species-poor regions (e.g. climate), the slowing of rates of biodiversity loss (and particularly of biotic homogenisation) during recent decades constitutes a positive sign indicating that, at least in regions where large land-use changes leading to natural habitat loss have nearly stopped, conservation efforts may be paying off.
