# Supplementary material for: Species richness declines and biotic homogenisation have slowed down for NW-European pollinators and plants
Source: Ecol Lett. 2013 May 21;16(7):870–8. doi: 10.1111/ele.12121 (PMC3738924; doi:10.1111/ele.12121)
Supplement: Supplementary file 1 [file ele0016-0870-SD1.pdf]

Supporting Information

"Species richness declines and biotic homogenization have slowed for NW European pollinators and plants" by Carvalheiro et al.

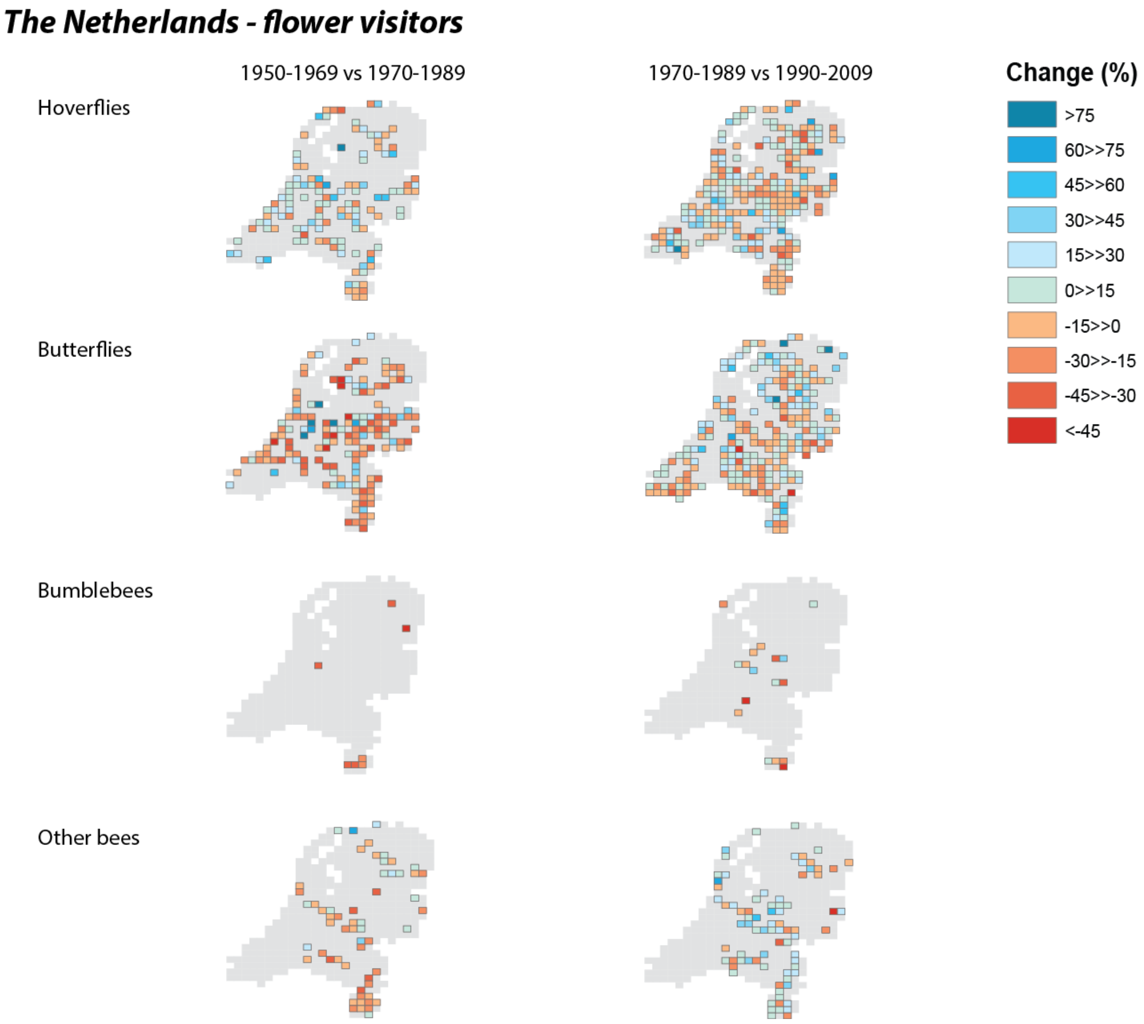

Figure S1.1 –Maps of richness change for 10km grid cells – flower visitors of the Netherlands.

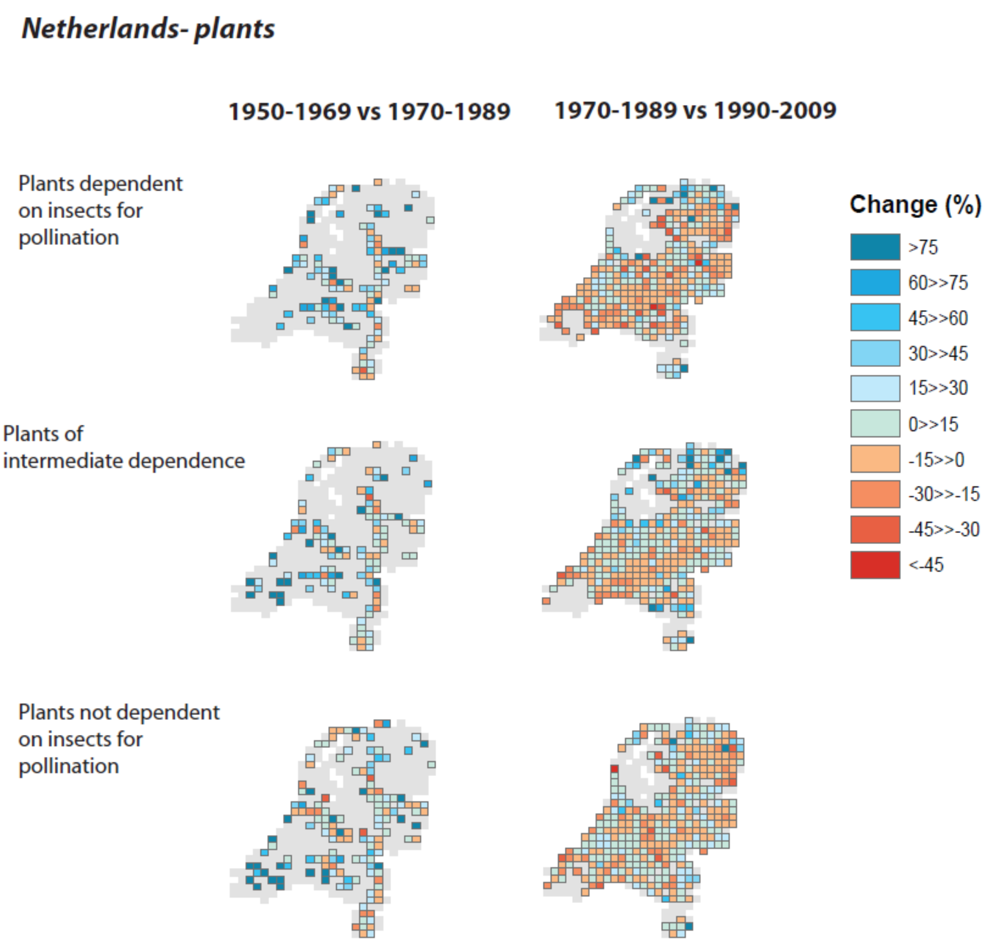

Figure S1.2 –Maps of richness change for 10km grid cells – plants of the Netherlands.

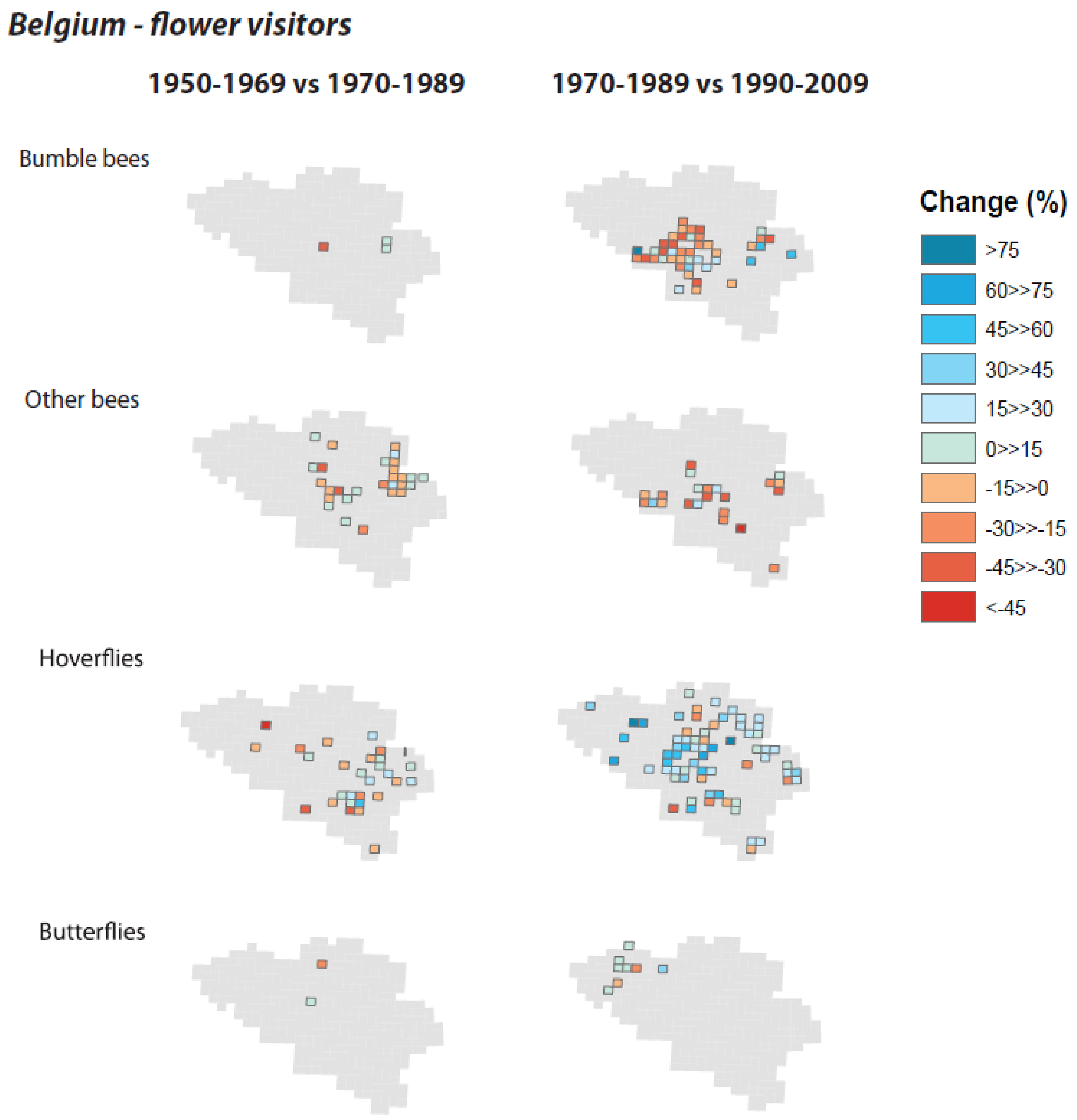

Figure S1.3 –Maps of richness change for 10km grid cells – flower visitors of Belgium.

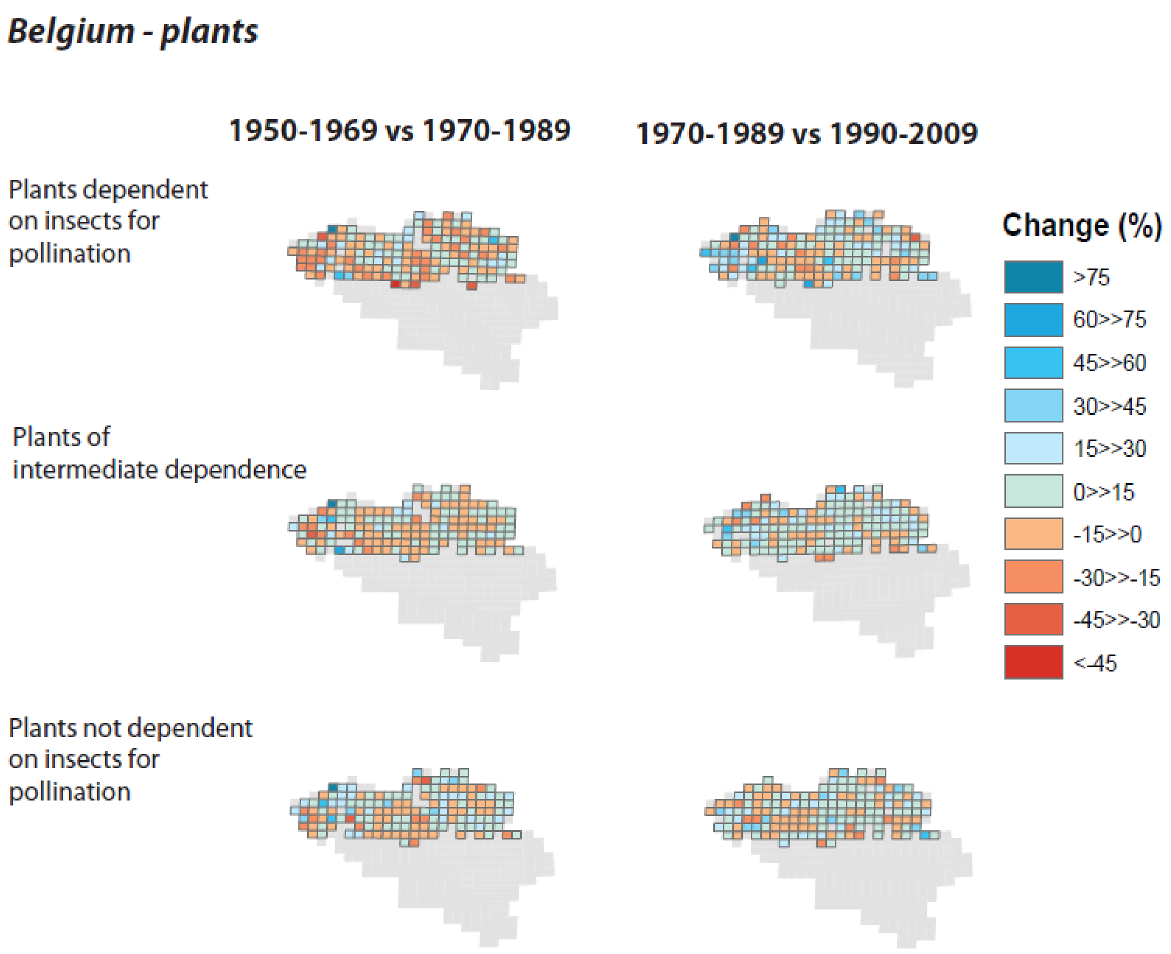

Figure S1.4 –Maps of richness change for 10km grid cells – plants of Belgium.

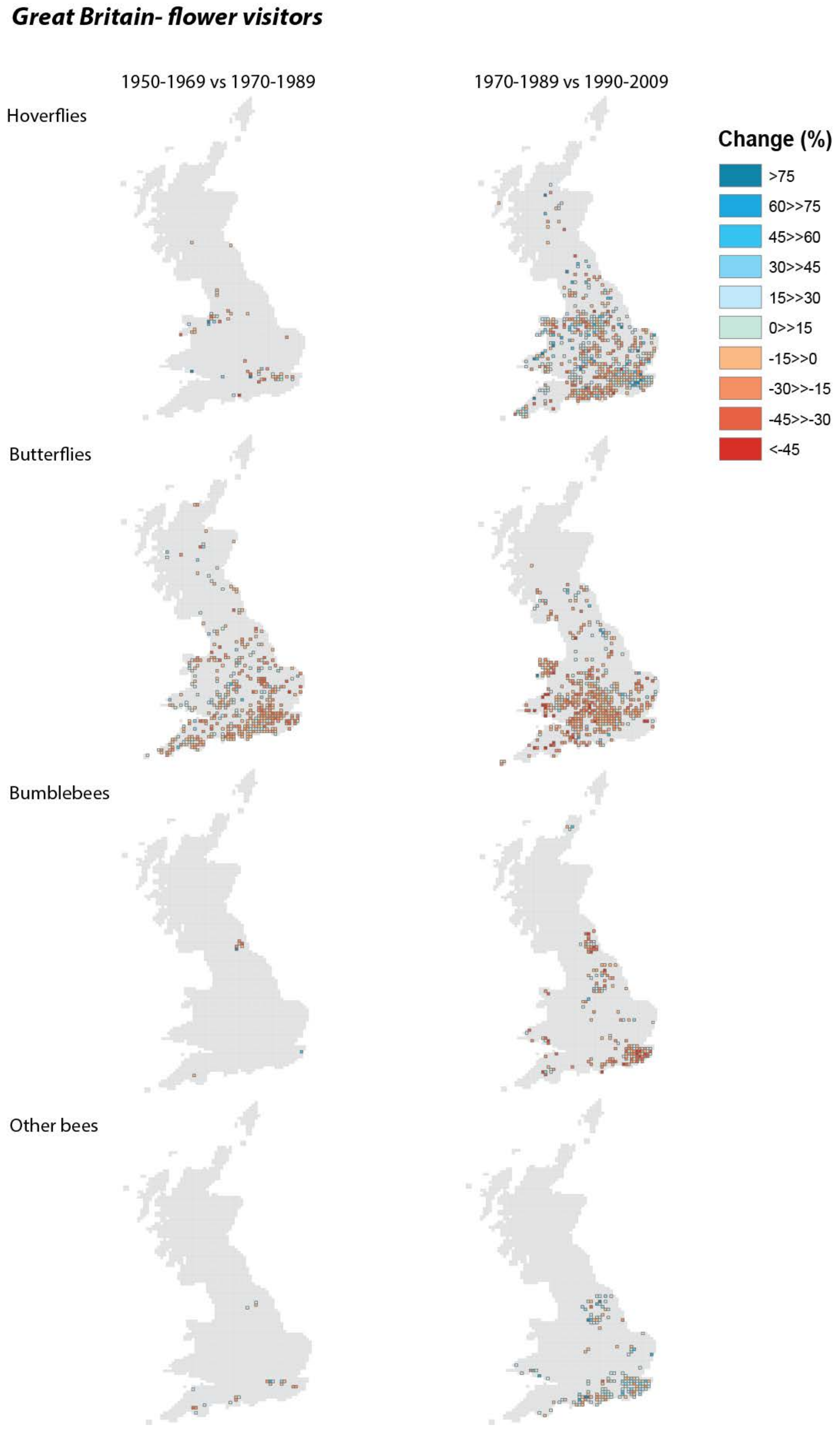

Figure S1.5 –Maps of richness change for 10km grid cells – flower visitors of Great Britain.

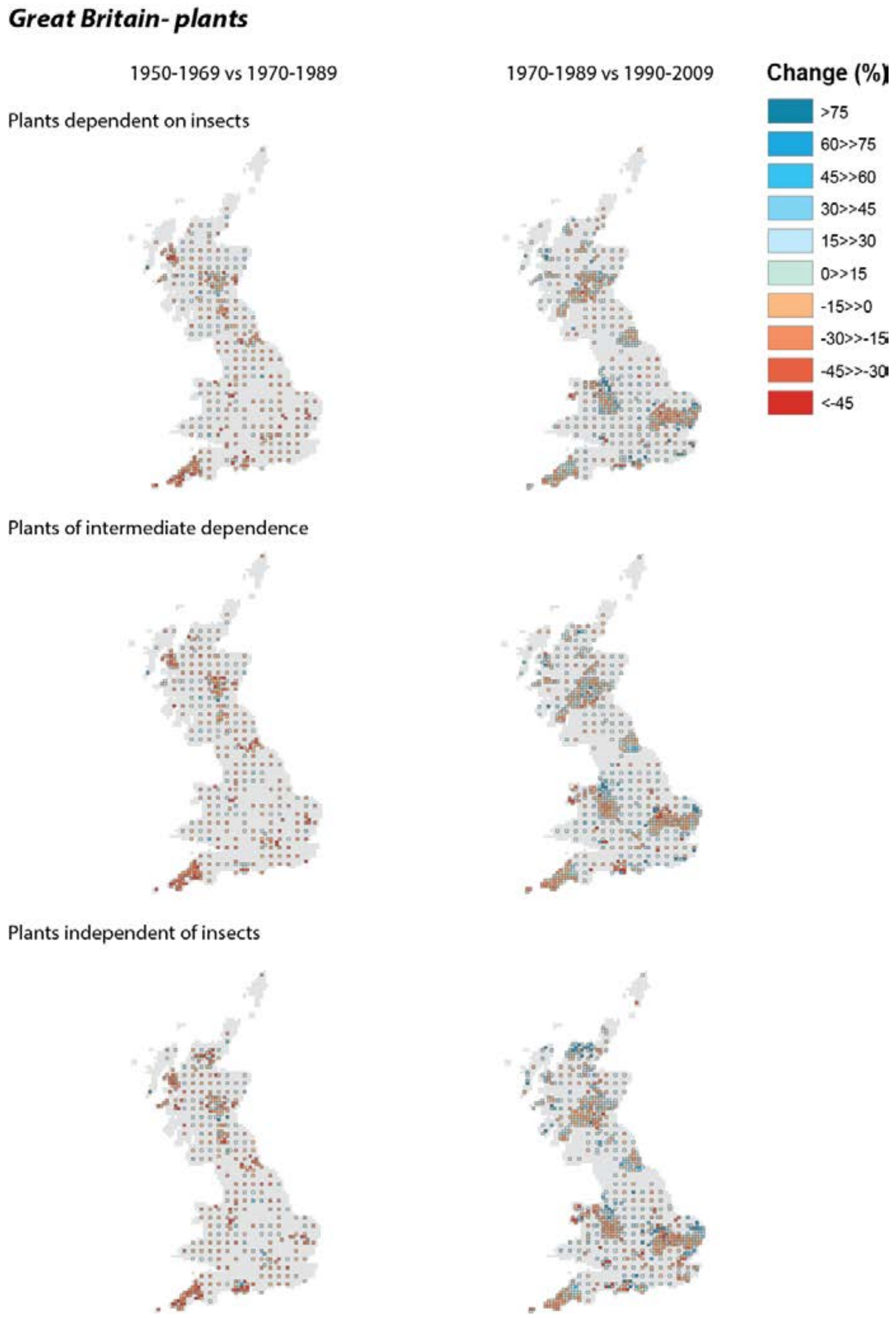

Figure S1.6 Maps of richness change for 10km grid cells – plants of Great Britain.

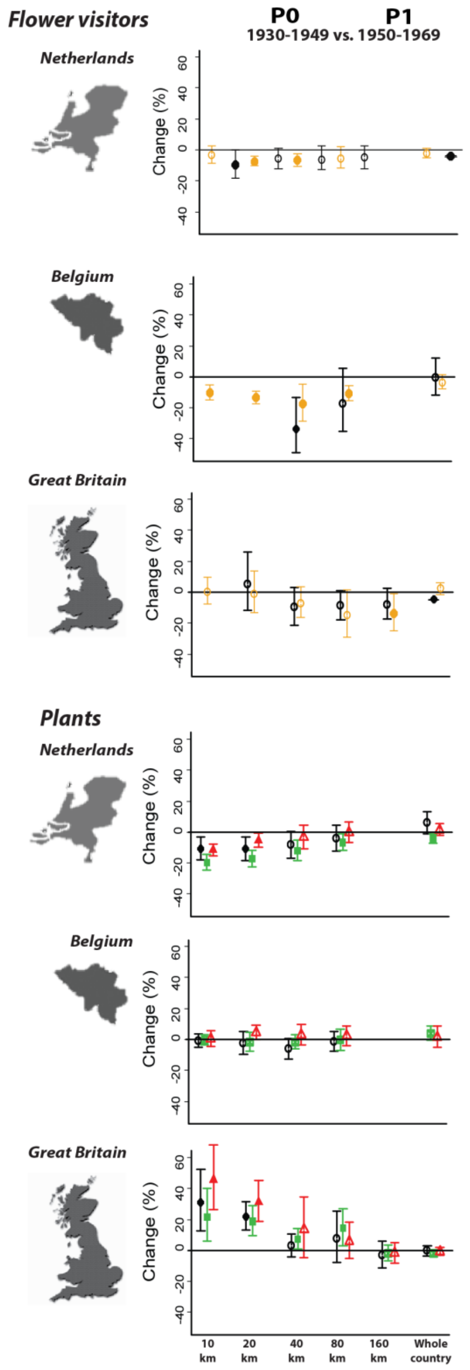

Figure S2 Change of native plant and bee species richness (estimated weighted mean ± 95% confidence intervals) **between P0 and P1 (i.e. 1930-1949 vs. 1950-1969)** through time at different spatial scales in Great Britain, the Netherlands and Belgium. As in the results presented in Figure 1, richness estimates were obtained using extrapolation ad interpolation (i.e. extrapolation was only allowed up to three times the number of records of the least sampled period). Change estimates (logratio) are back-transformed and presented as percentage of change. Horizontal line represents no change (0%). Filled symbols indicate that change was significantly different from zero, otherwise symbols are open (see statistical details in Table S2).

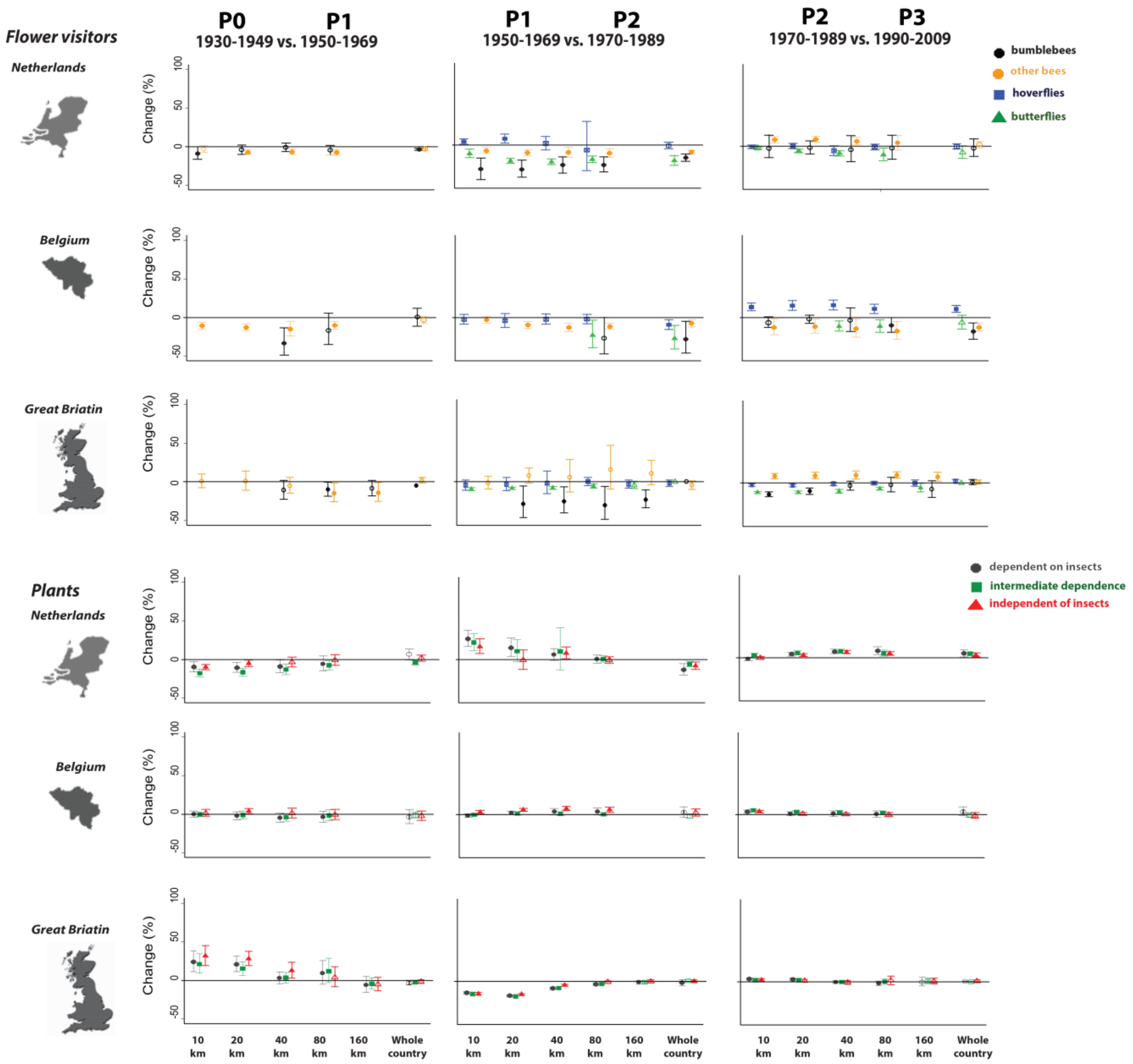

Figure S3 Change of species richness (estimated weighted mean ± 95% confidence intervals) of plants and flower visitors through time at different spatial scales in Great Britain, the Netherlands and Belgium, estimated using interpolation (rarefaction) of richness values. Change estimates (logratio) are back-transformed and presented as percentage of change. Horizontal line represents no change (0%). Filled symbols represent that change was significantly different from zero, otherwise symbols are open. Although evenness of records per species significantly changed with sampling effort and time period (Table S4, which affect richness change estimations per cell), overall we found consistent patterns of average richness change per spatial scale when using only interpolation.

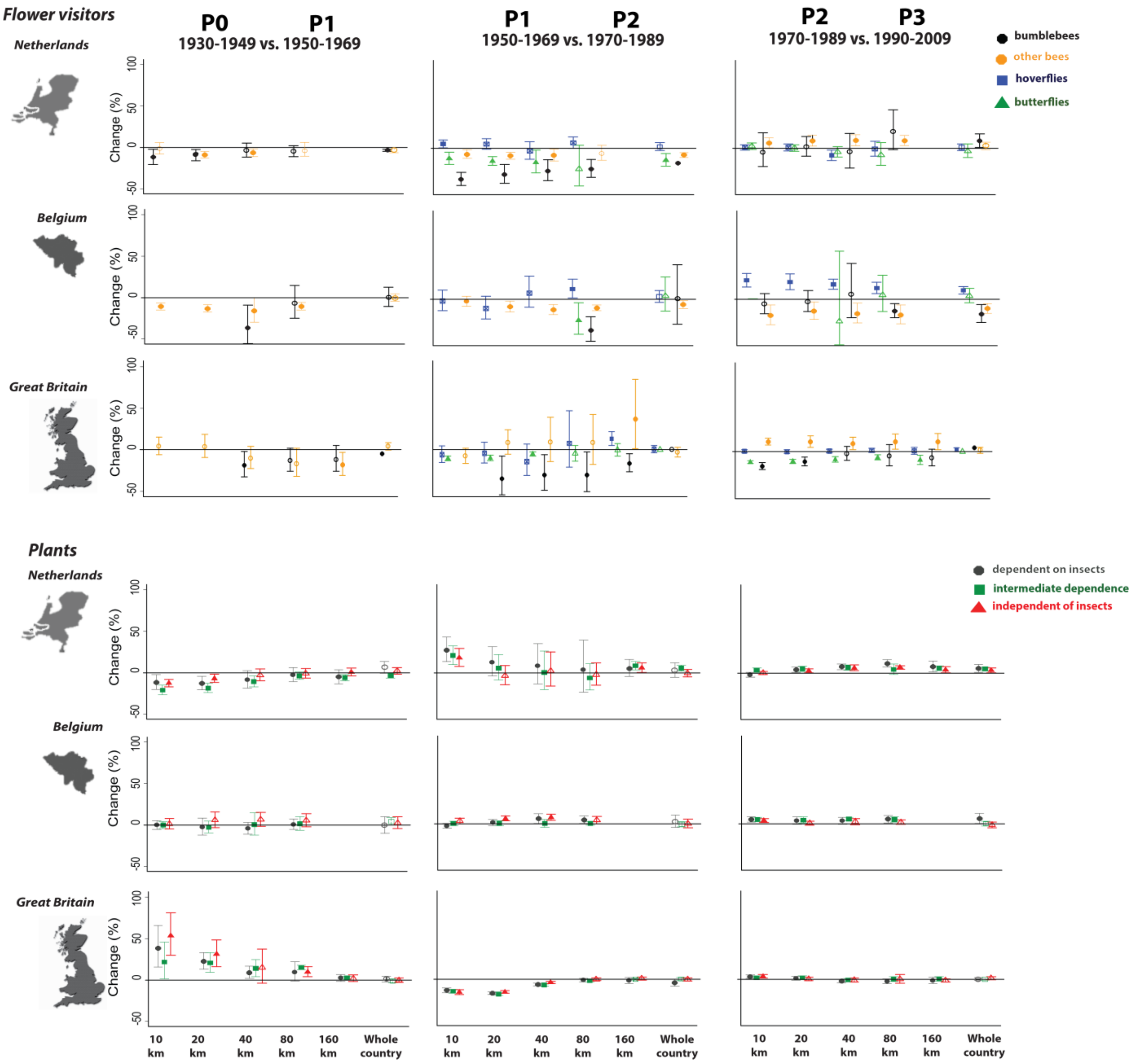

Figure S4. Change of species richness (estimated weighted mean ± 95% confidence intervals) of plants and flower visitors through time at different spatial scales in Great Britain, the Netherlands and Belgium, estimated using extrapolation of richness values. Change estimates (logratio) are back-transformed and presented as percentage of change. Horizontal line represents no change (0%). Filled symbols represent that change was significantly different from zero, otherwise symbols are open. Overall we found consistent patterns of average richness change per spatial scale when using only extrapolation.

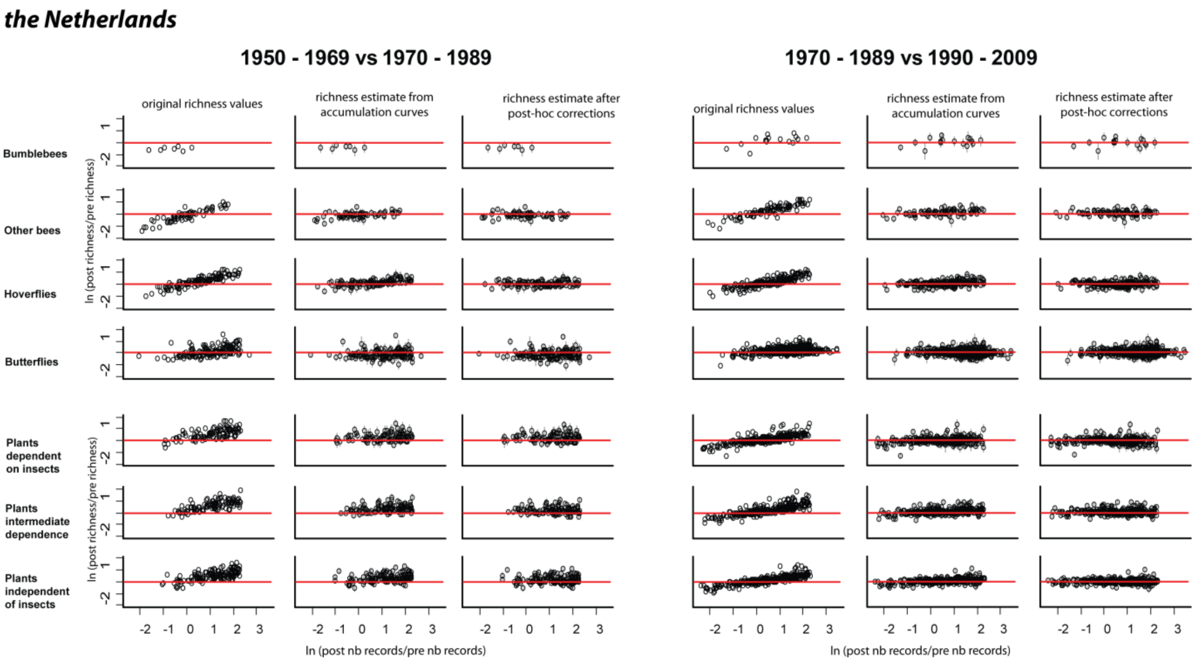

Figure S5.1 - Variation of values of relative richness change per gridcell ($ln\left[\frac{X_2(n)}{X_1(n)}\right]$) with relative sampling effort change ($ln\left[\frac{number\ of\ Records_2}{number\ of\ Records\ 1}\right]$) for the Netherlands. For each taxon within each period we present three graphs of the estimated richness change per 10km cell: based on original values; based on estimation from accumulation curves; based on estimation of change after post-hoc correction. Original values have a strong bias more species than expected by chance being detected in the period where there is a higher sampling effort (1st column) ; whenever accumulation curves did not fully correct for such bias a post-hoc correction was applied. Error bars represent the 95% Confidence Interval. We found that estimations based on accumulation curves removed most of the bias due to sampling effort, but estimates of richness change were sometimes still significantly positively correlated with the relative difference in sampling effort.

Belgium

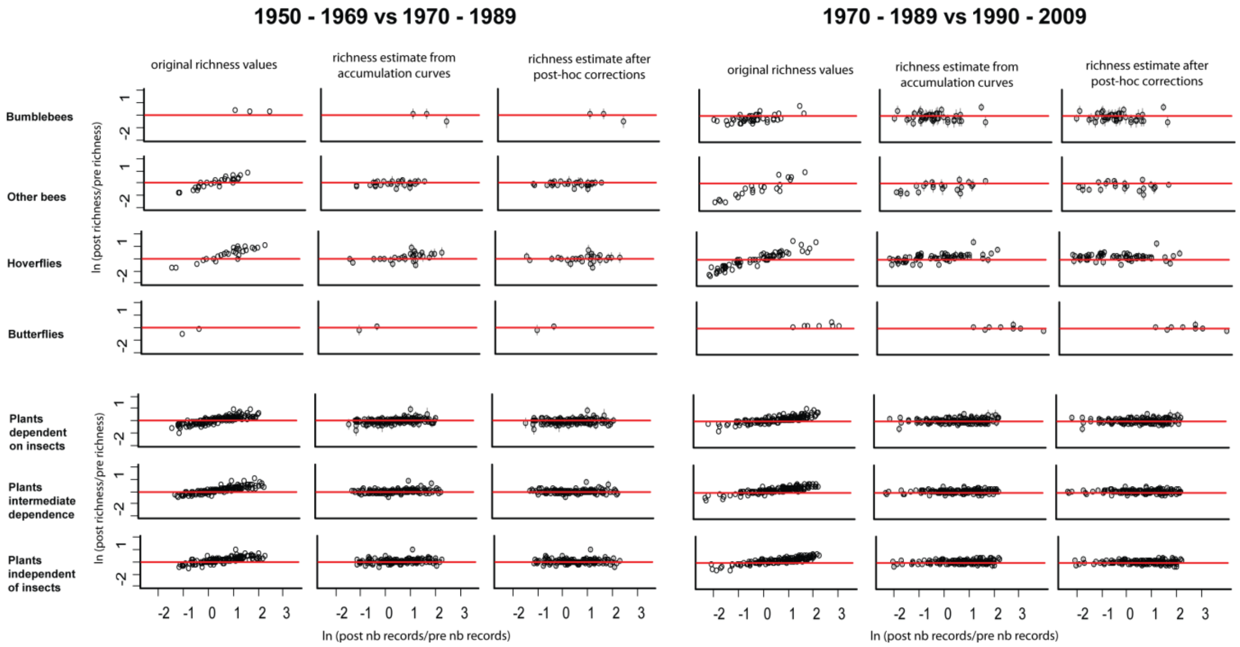

Figure S5.2 Variation of values of relative richness change per gridcell ($ln\left[\frac{X_2(n)}{X_1(n)}\right]$) with relative sampling effort change ($ln\left[\frac{number\ of\ Records_2}{number\ of\ Records_1}\right]$) for Belgium. For each taxon within each period we present three graphs of the estimated richness change per 10km cell: based on original values; based on estimation from accumulation curves; based on estimation of change after post-hoc correction. Original values have a strong bias more species than expected by chance being detected in the period where there is a higher sampling effort (1st column) ; whenever accumulation curves did not fully correct for such bias a post-hoc correction was applied. Error bars represent the 95% Confidence Interval. We found that estimations based on accumulation curves removed most of the bias due to sampling effort, but estimates of richness change were sometimes still significantly positively correlated with the relative difference in sampling effort.

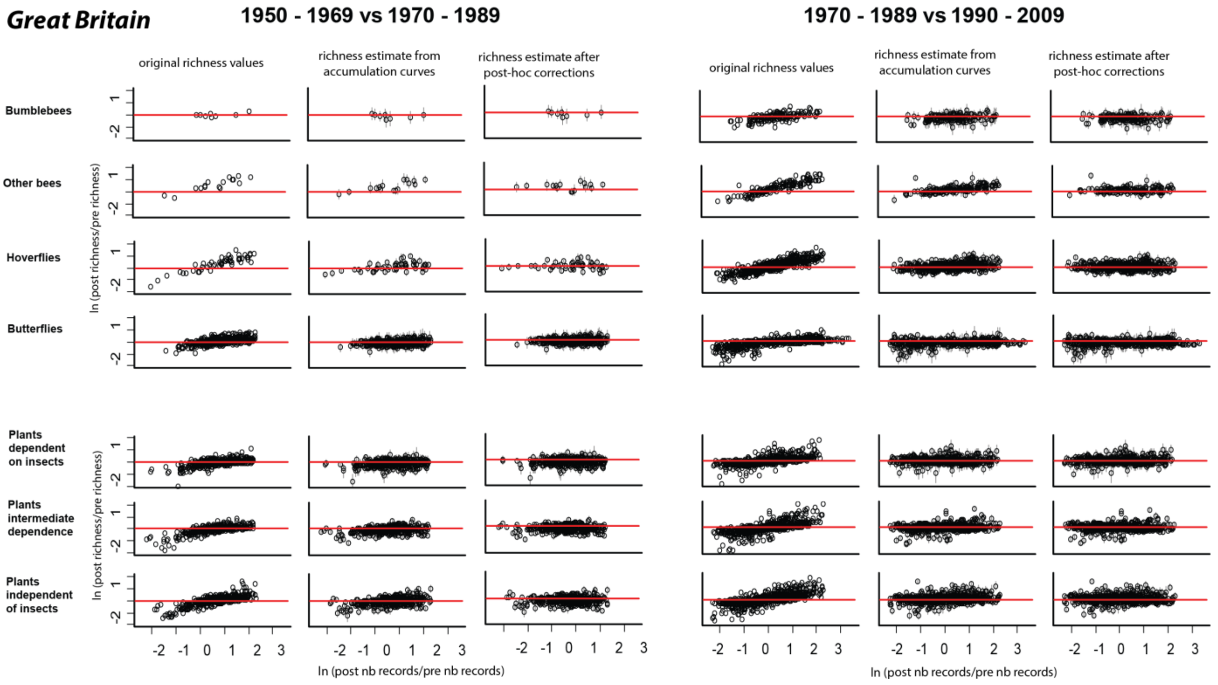

Figure S5.3 Variation of values of relative richness change per grid cell ($ln\left[\frac{X_2(n)}{X_1(n)}\right]$) with relative sampling effort change ($ln\left[\frac{number\ of\ Records_2}{number\ of\ Records\ 1}\right]$) for Great Britain. For each taxon within each period we present three graphs of the estimated richness change per 10km cell: based on original values; based on estimation from accumulation curves; based on estimation of change after post-hoc correction. Original values have a strong bias more species than expected by chance being detected in the period where there is a higher sampling effort (1st column) ; whenever accumulation curves did not fully correct for such bias a post-hoc correction was applied. Error bars represent the 95% Confidence Interval. We found that estimations based on accumulation curves removed most of the bias due to sampling effort, but estimates of richness change were sometimes still significantly positively correlated with the relative difference in sampling effort.

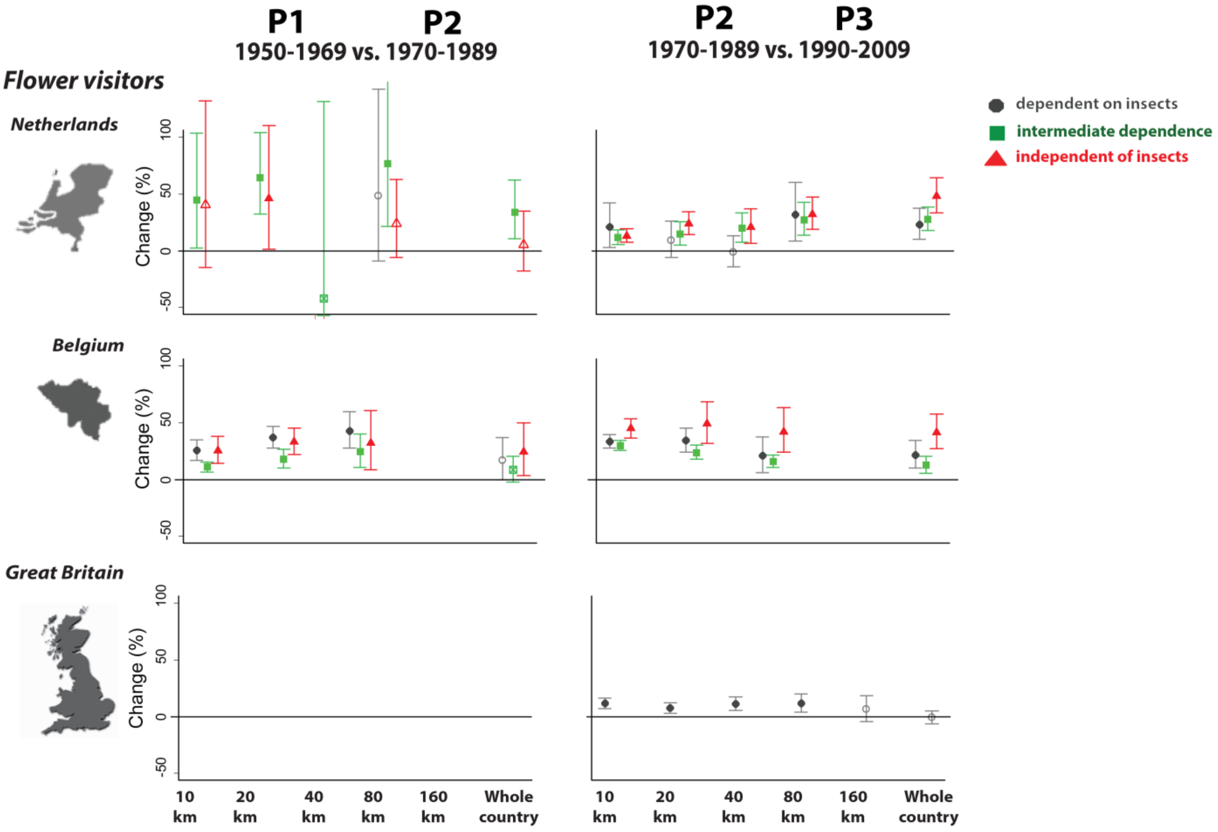

Figure S6 Change of exotic plants (estimated weighted mean ± 95% confidence intervals) through time at different spatial scales in Great Britain, the Netherlands and Belgium. As in the results presented in Fig. 1, richness estimates were obtained using extrapolation and interpolation (i.e. extrapolation was only allowed up to three times the number of records of the least sampled period). Change estimates (logratio) are back-transformed and presented as percentage of change for the spatial scales and countries for which data quality allowed. Horizontal line represents no change (0%). Filled symbols indicate that change was significantly different from zero, otherwise symbols are open.

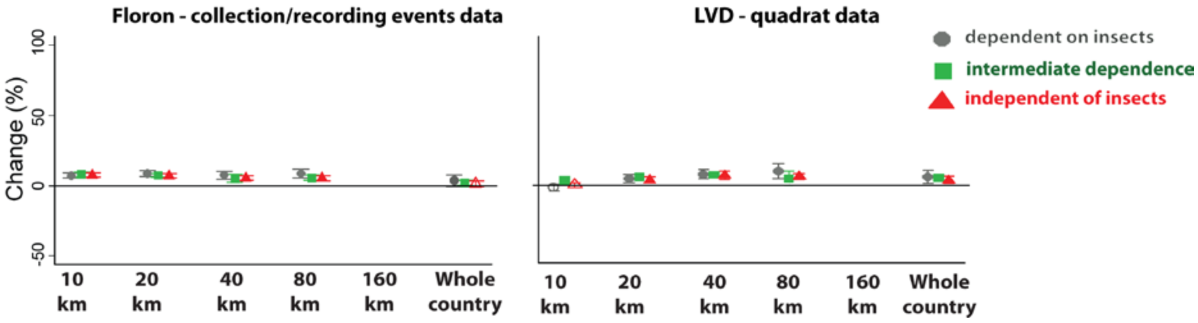

Figure S7 Estimate of change of Dutch native plant (estimated weighted mean ± 95% confidence intervals) between 1970-1989 vs 1990-2009 at different spatial scales using two different methods: quadrat surveys (LVD database) and collection/recording events (FLORON database). NDFF FLORON database is composed of records from collection and recording events (data for this project managed by BO). As in the results presented in Figure 1, richness estimates were obtained using extrapolation and interpolation (i.e. extrapolation was only allowed up to three times the number of records of the least sampled period). Change estimates (logratio) are back-transformed and presented as percentage of change. Horizontal line represents no change (0%). Filled symbols indicate that change was significantly different from zero, otherwise symbols are open. For most spatial scales (20km up to whole country) results found with the two types of databases were similar.

Table S1. Details for each taxonomic group dataset. For plants and butterflies of Belgium only Flanders for the Northern region was used. For plants of the Netherlands the numbers within the table correspond to data from quadrat surveys (LVD, available through all time periods), which were used for the analyses presented in the main text, and confirmed the results after 1970s using available collection/recording event data available from FLORON (3 841 884 records for 1970-1989 and 6 876 452 records for 1990-2009). For Great Britain, bee data were obtained from BWARS – the Bees, Wasps and Ants Recording Society (data for this project managed by SPMR), hoverfly data from BRC-Biological Records Centre, butterfly data from Butterfly Conservation (data for this project managed by RF); and plant data were obtained from the Botanical Society of the British Isles' Vascular Plant Database (data for this project managed by QG). Belgian bee data were obtained from the Banque de données fauniques Gembloux-Mons (BDFGM) (data for this project managed by PR and DM), hoverfly data were managed by FVM, butterfly data from the joint butterfly database of INBO and the Butterfly Working Group of Natuurpunt (data for this project managed by DM) and plant data from Florabank* (data for this project managed by WVL). For the Netherlands bee and hoverfly data were obtained from European Invertebrate Survey (EIS) - the Netherlands (data for this project managed by MR), butterfly data were obtained from the Dutch National Database of Flora and Fauna (NDFF, www.natuurloket.nl) (data for this project managed by MFW) and plant data were obtained from *Landelijke Vegetatie Databank* - LVD, from the Dutch National Vegetation Database (http://www.synbiosys.alterra.nl/lvd) (survey data for this project managed by JS and SH). Information on the dependency of plants on insects for pollination was obtained from the Ecological Flora database, ECOFLOR, BIOFLOR and BIOBASE **. Databases were obtained/accessed in March 2011. For plants, only native species are considered in this table. As an indication of current susceptibility of the studied taxa we provide an indication of vulnerable species of considerable conservationist interest (encompassing species classified as critically endangered to nearly threatened according to Red List categories or taxa-specialist criteria ***) species.

Group	Total number of specimens and species in the databases						% current, i.e. not extinct. vulnerable species (total number)
	'50-'69		'70-'89		'90-'09		
	specimens	Species	specimens	Species	specimens	Species	
Great Britain							
Plants exclusively pollinated by insects	95 497	104	174 613	102	143 838	102	25 %
Plants partially pollinated by insects	479 195	492	778 935	498	649 538	496	of the 1747
Plants not pollinated by insects	281 796	321	518 727	323	395 784	324	native sps
Other bees	4 573	196	44 105	204	147 773	209	30% of the 223 sp
Bumble bees	1 648	21	17 786	21	71 203	22	50 % of the 24 sps
Hoverflies	15 292	243	213 759	260	318 059	268	18% of the 268 sps
Butterflies	48 717	58	245 314	58	1 271 507	58	52% of the 58 sps
The Netherlands							
Plants exclusively pollinated by insects	28 970	152	464 775	165	414 674	173	27%
Plants partially pollinated by insects	96 545	515	1 532 717	565	1 292 203	590	of the 1376
Plants not pollinated by insects	94 659	300	1 516 257	325	1 554 834	336	native sps
Other bees	20 268	274	28488	253	84 630	267	30% of the 316 sps
Bumble bees	3 252	28	1 914	23	14 856	22	65% of the 23 sps
Hoverflies	39 701	271	121 424	288	263 302	298	28% of the 316 sps
Butterflies	29 496	91	162 102	79	1 835 545	84	37% of the 84 sps (migrants included)
Belgium							
Plants exclusively pollinated by insects	27 003	112	56 271	119	98 387	128	22%
Plants partially pollinated by insects	98 162	456	207 938	466	410 999	478	of the 1110
Plants not pollinated by insects	53 773	250	125 218	269	217 439	272	native sps(only Flanders)
Other bees	9 947	322	13 358	325	5 608	262	12% of the 330 sps
Bumble bees	438	24	6 584	25	2810	19	20% of the 25 sps
Hoverflies	7192	258	67 025	291	38 283	307	36% of the 328sps
Butterflies	357	48	9 040	52	601 383	55	52% of the 51 sps (only Flanders, migrants included) (4 extinct btw 1990-2009)

* Van Landuyt, W. Vanhecke, L., Brosens, D. (2012). Florabank 1: a grid-based database on vascular plant distribution in the northern part of Belgium (Flanders and the Brussels Capital region). *PhytoKeys*, 12, 59–67.
** Fitter, A . H., Peat , H. J. (1994). The ecological flora database. *J. Ecol*. 82, 415-425.
Klotz, S., Kühn, I., Durka, W. (2002). *BIOLFLOR - Eine Datenbank zu biologisch-ökologischen Merkmalen der Gefäßpflanzen in Deutschland. - Schriftenreihe für Vegetationskunde* 38. Bundesamt für Naturschutz. [http://www2.ufz.de/biolflor/index.jsp] Last accessed 15 December 2012.
BioBase. (2003). *Natuur in Cijfers*. [CBS/RIVM/Stichting DLO.]
*** Bos FG, Bosveld MA, Groenendijk DG, Van Swaay CAM, Wynhoff I (2006) De Dagvlinders van Nederland - Verspreiding en Bescherming. Nederlandse Fauna 7. Nationaal Natuurhistorisch Museum Naturalis, KNNV Uitgeverij & EIS-Nederland, Leiden.
Dines, T.D., Jones, R.A., Leach, S.J., McKean, D.R., Pearman, D.A., Preston, C.D., Rumsey, F.J. & Taylor I. (2005) *Species status No 7: The vascular plant red list of Great Britain.* Joint Nature Conservation Committee Cheffings, C.M. & Farrell, L. (Eds) URL: http://jncc.defra.gov.uk/pdf/pub05_speciesstatusvpredlist3_web.pdf
Fox, R., Warren, M.S., Brereton, T.M., Roy, D.B. and Robinson, A. (2011). A new red list of British butterflies. *Insect Conservation and Diversity* **4**, 159-172
Maes D et al. 2012. Applying IUCN Red List criteria at a small regional level: A test case with butterflies in Flanders (north Belgium). *Biological Conservation* 145: 258-266.
Peeters, T.M.J. & M. Reemer 2003. Bedreigde en verdwenen bijen in Nederland (Apidae s.l.). Basisrapport met voorstel voor de Rode Lijst. - EIS-Nederland, Leiden.
Rasmont, P., J.Leclercq, A.Jacob-Remacle, A.Pauly & C.Gaspar, 1993. *The faunistic drift of Apoidea in Belgium*. pp. 65-87 in E. Bruneau, *Bees for pollination*, Commission of the European Communities,
Brussels, 237 pp.
Reemer, M., W. Renema, W. van Steenis, T. Zeegers, A. Barendregt, J.T. Smit, M.P. van Veen, J. van Steenis & L.J.J.M. van der Leij 2009. De Nederlandse zweefvliegen (Diptera: Syrphidae). - Nederlandse Fauna 8: 1-442.
Sullivan, M.S., Gilbert, F., Rotheray, G., Croasdale,S. & Jones, M. (2000) Comparative analyses of correlates of Red data book status: a case study using European hoverflies (Diptera: Syrphidae). *Animal Conservation* 3, 91–95.
Shirt, D. B. (1987). *British Red Data Books 2. Insects*, Nature Conservancy Council, Peterborough (UK)
Falk, S. (1991). *A Review of the Scarce and Threatened Bees, Wasps and Ants of Great Britain.* Report of Nature Conservancy Council, Peterborough (UK).

Tables S2-S6: see attached Excel file

Supplementary Table captions

Table S2. Statistical details of richness change analyses. Richness change was calculated based on estimates of richness using interpolation and extrapolation, and log transformed values (logratio) were analyzed with a weighted General Linear Models (GLMw), using the inverse of variance (bootstrapped to correct for under/over-representation of singletons) as weight. For richness analyses, exclusion of poor quality cells was based on criteria that minimized sensitivity of the analyses to the removal of single grid-cell values: we excluded grid-cells with sampling effort lower than 20% of the maximum number of species found per cell (for low diversity groups, 15 was the minimum number of records per cell allowed) and with a ratio of records/number of species higher than 1.5. Also, unless sampling effort of the least sampled period was very high (*i.e.* more than five-fold the maximum number of species found in that country), we excluded grid-cells with a difference in number of records between the two time periods larger than 10-fold (see also Biesmeijer *et al.* 2006; Keil *et al.* 2011).

Table S3. Statistical details of effect of distance between cells and time period on species assemblage similarity analyses. Similarity values (β-sim) were logit transformed to normalize residuals and analyzed with General Linear Mixed Models (GLMM) with grid cell within time period as random variable, and sampling effort, distance and time period and its interaction as fixed terms. Seff - sampling effort of least sampled cell; Dif - Difference in sampling effort; T*D - interaction Time period and Distance. For similarity analyses we selected only 10km grid cells which had record numbers equal to or higher than the maximum number of species possible to find in each cell (instead of 20% of that maximum number), and which had a ratio of records/number of species higher than 1.5.

Table S4 . Effect of sampling effort and time period on evenness of records per species. Both sampling effort and evenness were log-transformed to normalize residuals and analyzed with GLMM, with grid cell within time period as random variable.

Table S5. Sensitivity of richness change analyses to weights. Richness change was calculated based on estimates of richness using interpolation and extrapolation, and analyzed with GLMw, with three different types of weights: B - the inverse of variance (bootstrapped), NB - non bootstrapped inverse of variance, and UN - unweighted. NL - Netherlands; BL - Belgium; GB - Great Britain.

Table S6. Evaluation of spatial autocorrelation of richness change values of plants and flower visitors in the Netherlands and Belgium Great Britain. Model with spatial autocorrelation structure (Linear or Exponential) were compared with a null model using a

log-likelihood ratio test. Akaike Information Criterion values are provided for each model. CS - Correlation structure.
